# Shoulder and Elbow Surgery Special Issue

**DOI:** 10.1111/os.13861

**Published:** 2023-08-16

**Authors:** Marius M. Scarlat, Xiaoming Wu, Yuren Du

**Affiliations:** ^1^ Clinique Chirurgicale St Michel, Grope ELSAN Toulon France; ^2^ Department of Orthopaedics and Traumatology Shanghai General Hospital affiliated to Shanghai Jiao Tong University Shanghai Shanghai China; ^3^ Editorial Office of Orthopaedic Surgery Tianjin Hospital Tianjin China

It is a pleasure and honor to present the editorial of this special issue dedicated to shoulder and elbow surgery.

*Orthopaedic Surgery* is an uprising star in the constellation of specialized scientific medical journals, with its performance, visibility, and downloads growing, and a huge list of subscribers and many submissions from China and worldwide.

The success of our previous special issue was noted—it brought a high number of views, downloads, and citations. Therefore, in this new special issue we decided to bring in new subjects of research, techniques, clinical and experimental research. These articles were all peer‐reviewed and amended according to the observations and comments of our expert reviewers, who we would like to acknowledge here.

The three‐year pandemic restricted not only inter‐city and international travel; it also had an impact on academic activities and publications. To this end, as guest editors we decided to publish a special issue on hot topics in the field of shoulder and elbow surgery. This issue features 32 professional articles, including three review articles, 21 clinical articles, four research articles, two operative techniques, and two case reports, contributed by well‐known orthopaedic experts from China, the United States, Korea, and Europe.

The elbow section of this issue includes papers on arthroscopy,^1^ open surgery in elbow stiffness^2^, ^3^ fractures,^4^ total elbow arthroplasty,^5^ and conservative methods of treatment.^6^ The shoulder section addresses a multitude of anatomic details as well as techniques, including clavicular shaft and distal fractures, acromioclavicular joint traumatic and degenerative conditions,^7^, ^8^, ^9^, ^10^, ^11^ rotator cuff pathologies and fixation techniques, treatment of the post‐surgical or post‐traumatic glenohumeral stiffness, fractures of the proximal humerus,^5^, ^12^, ^13^, ^14^, ^15^, ^16^, ^17^, ^18^, ^19^, ^20^, ^21^ the safety of irrigation in the shoulder arthroscopy, management of the scapular and glenoid fractures, radiologic and 3D models.^22^, ^23^, ^24^, ^25^, ^26^, ^27^, ^28^, ^29^, ^30^, ^31^

Overall, this special issue contains plenty of meaningful readings for surgeons specializing in shoulder and elbow surgery and for general orthopaedic and trauma specialists, as well as for orthopaedic residents and young surgeons worldwide. Enjoy reading, sharing the data and the wonderful illustrations from this special issue, and we look forward to receiving your feedback.
